# High performance dengue virus antigen-based serotyping-NS1-ELISA (plus): A simple alternative approach to identify dengue virus serotypes in acute dengue specimens

**DOI:** 10.1371/journal.pntd.0009065

**Published:** 2021-02-26

**Authors:** Tanapan Prommool, Pongpawan Sethanant, Narodom Phaenthaisong, Nattaya Tangthawornchaikul, Adisak Songjaeng, Panisadee Avirutnan, Dumrong Mairiang, Prasit Luangaram, Chatchawan Srisawat, Watchara Kasinrerk, Sirijitt Vasanawathana, Kanokwan Sriruksa, Wannee Limpitikul, Prida Malasit, Chunya Puttikhunt

**Affiliations:** 1 Molecular Biology of Dengue and Flaviviruses Research Team, Medical Molecular Biotechnology Research Group, National Center for Genetic Engineering and Biotechnology, National Science and Technology Development Agency, Bangkok, Thailand; 2 Division of Dengue Hemorrhagic Fever Research, Faculty of Medicine Siriraj Hospital, Mahidol University, Bangkok, Thailand; 3 Siriraj Center of Research Excellence in Dengue and Emerging Pathogens, Faculty of Medicine Siriraj Hospital, Mahidol University, Bangkok, Thailand; 4 Department of Biochemistry, Faculty of Medicine Siriraj Hospital, Mahidol University, Bangkok, Thailand; 5 Biomedical Technology Research Center, National Center for Genetic Engineering and Biotechnology, National Sciences and Technology Development Agency, Chiang Mai, Thailand; 6 Division of Clinical Immunology, Department of Medical Technology, Faculty of Associated Medical Sciences, Chiang Mai University, Chiang Mai, Thailand; 7 Khon Kaen Hospital, Khon Kaen, Thailand; 8 Songkhla Hospital, Songkhla, Thailand; DoD - AFHSB, UNITED STATES

## Abstract

Dengue hemorrhagic fever (DHF) is caused by infection with dengue virus (DENV). Four different serotypes (DENV1-4) co-circulate in dengue endemic areas. The viral RNA genome-based reverse-transcription PCR (RT-PCR) is the most widely used method to identify DENV serotypes in patient specimens. However, the non-structural protein 1 (NS1) antigen as a biomarker for DENV serotyping is an emerging alternative method. We modified the serotyping-NS1-enzyme linked immunosorbent assay (stNS1-ELISA) from the originally established assay which had limited sensitivity overall and poor specificity for the DENV2 serotype. Here, four biotinylated serotype-specific antibodies were applied, including an entirely new design for detection of DENV2. Prediction of the infecting serotype of retrospective acute-phase plasma from dengue patients revealed 100% concordance with the standard RT-PCR method for all four serotypes and 78% overall sensitivity (156/200). The sensitivity of DENV1 NS1 detection was greatly improved (from 62% to 90%) by the addition of a DENV1/DENV3 sub-complex antibody pair. Inclusive of five antibody pairs, the stNS1-ELISA (plus) method showed an overall increased sensitivity to 85.5% (171/200). With the same clinical specimens, a commercial NS1 rapid diagnostic test (NS1-RDT) showed 72% sensitivity (147/200), significantly lower than the stNS1-ELISA (plus) performance. In conclusion, the stNS1-ELISA (plus) is an improved method for prediction of DENV serotype and for overall sensitivity. It could be an alternative assay not only for early dengue diagnosis, but also for serotype identification especially in remote resource-limited dengue endemic areas.

## Author summary

Four serotypes of DENV co-circulate in dengue endemic areas. Secondary infection with a different DENV serotype is beleived to involve with severe dengue disease. Standard laboratory diagnosis to identify DENV serotypes in dengue patient specimens is performed by sophisticated genome-based RT-PCR method with serotype-specific oligoprimers. We have previously established an alternative protein-based NS1 assay for DENV serotyping namely, a serotyping-NS1-ELISA (stNS1-ELISA), with the use of serotype-specific monoclonal antibodies (Mabs) to NS1 protein. Due to its unsatisfactory performance, the stNS1-ELISA was modified in this study. The biotinylated serotype-specific detection Mabs were introduced to enhance the overall sensitivity. A new DENV2-specific antibody was applied to improve DENV serotype identification. Prediction of infecting serotype from NS1-positive samples by our modified assay was 100% concordant with the standard RT-PCR method for all four serotypes. The overall sensitivity was greatly improved by an additional DENV1/DENV3 sub-complex antibody. This modified assay is efficient not only for early dengue diagnosis, but also for serotype identification in epidemiological studies and disease surveillance.

## Introduction

Dengue virus (DENV), the cause of the mosquito-borne disease dengue hemorrhagic fever (DHF), comprises four different serotypes (DENV1, DENV2, DENV3 and DENV4) co-circulating in tropical and subtropical endemic areas worldwide. The incidence of dengue has dramatically increased over the past 50 years, both in the number of cases and countries affected by this disease. About 390 million people worldwide are infected each year, of which 96 million present with clinical symptoms [1]. Secondary infection by heterologous serotypes of DENV cause more severe DHF cases than primary infection [2], possibly as a result of the antibody-dependent enhancement (ADE) phenomenon [3]. Sequential infection by certain serotypes were found to contribute to disease severity in some populations [4–7]. For example, in Thailand hospital cohort (1994–2005), dengue serotype infection sequences leading to DHF were documented in DENV1 followed by DENV4 or DENV2 followed by DENV3, or DENV3 followed by DENV1, or DENV3 followed by DENV4. [6]. In Cuba (1981) and Havana (2002–2002), reports indicate that DHF occurs more frequently in DENV-1 exposed individuals followed with DENV-2 or DENV-3 infections [4,7]. The increasing time between sequential infection was also associated with greater severity of the second detected infection [5] Serotypes of dengue virus were found to be associated with disease severity under certain conditions such as immune status, geographical areas and epidemic years. For examples, a meta-analysis of relevant dengue over 15,000 cases in 31 studies over the past 50 years was found that primary infection with DENV3 and secondary infection with DENV2, DENV3 and DENV4 increased the risk of dengue severity in Southeast Asia [8]. In Brazil (during 2009–2013), DENV2 infections were shown to be seven times more common among severe case patients than other serotypes [9]. In Singapore (during 2005–2011), DENV1 was associated with a relatively higher risk of severe DHF than DENV2 in adult dengue patients [10]. Although medical treatment or intervention by clinicians is the same irrespective of dengue infecting serotypes, data on which serotypes are prevalent among severe cases is still important for public health studies. Early and rapid detection of DENV transmission and current epidemic serotypes is critical for better disease surveillance and epidemiological control. Despite conventional virus isolation, laboratory diagnosis of DENV infection in patient specimens is currently performed using three different approaches, i.e. detection of DENV RNA genome (by reverse-transcription polymerase chain reaction, RT-PCR), DENV non-structural protein 1 (NS1) protein (by NS1 ELISA or rapid test) and antibody responses to DENV (by anti-dengue IgM/IgG ELISA or rapid test) [11]. DENV RNA and NS1 protein can be detected in the early phase of infection while patients still have high fever. DENV RNA decreases and clears from blood circulation just before or on the day of defervescence, but NS1 protein persists for a longer period than viral RNA [12–14]. Anti-DENV antibody responses are relatively higher at the late phase of infection. They are thus preferentially used to confirm clinical diagnosis of DENV infection. However, anti-DENV IgM antibody can be present during acute phase of infection (when NS1 antigen is still present) earlier than the late IgG isotypes, especially in primary infected cases. Therefore, with the combination of the early IgM antibody and NS1 antigen assays, the sensitivity of dengue diagnostic tests has increased [15–18]. Detection of viral genome by RT-PCR with serotype-specific primers shows high sensitivity and accuracy and has been adopted as a standard method to identify DENV serotypes; it has replaced virus isolation followed with serotype-specific antibody detection [19]. Nevertheless, the sensitivity of RT-PCR varies between 80–90% depending on primer sets and other factors [20, 21]. Although realtime or quantitative RT-PCR method is more sensitive, but it is complicated and costly, and requires a special laboratory and well-trained technicians, which is not suitable for resource-poor areas where dengue is endemic.

Detection of DENV NS1 antigen has become a widely used laboratory diagnostic test for DENV infection over the past 10 years. NS1 is secreted into the blood circulation during the febrile phase of the disease, making NS1 a promising early diagnostic marker. The levels of NS1 in the circulation have been reported to be between 10 ng/mL and 50 μg/mL [22] and correlate with disease severity [23–25]. At present, several dengue NS1 diagnostic kits are available on the market, both in capture ELISA and rapid diagnostic test (RDT) formats. However, most of them show unequal sensitivity among the four serotypes [15,16,26]. Several clinical evaluations of various NS1 diagnostic kits demonstrated a wide range of performance, which may be dependent on certain factors, for example, type of kit (ELISA or RDT), characteristics of tested specimens (immune status, date of collection), DENV serotypes (DENV1 to DENV4) and geographical areas (endemic or non-endemic) [15,27–29]. The NS1-RDT format is far more widely accepted than NS1-ELISA due to its simplicity and rapidity (results in 20–30 mins), although its overall sensitivity is generally lower than that of ELISA [26,30]. So far, none of commercial dengue NS1 tests can identify infecting serotypes of DENV-infected samples.

Due to the presence of distinct NS1 epitopes among the four serotypes [31], NS1 assays with the addition of serotype identification can be performed by using monoclonal antibodies specific to the NS1 of each serotype. NS1 capture ELISAs, which can differentiate the four DENV serotypes simultaneously, were reported in 2011 by our group [32] and by Ding et al [31] and more recently by others using different sets of monoclonal antibodies [33–35]. Although these assays showed high serotype specificity in the developmental phase, their clinical validation of dengue patient specimens were incomplete for some serotypes.

We previously developed the stNS1-ELISA to distinguish four DENV serotypes as an alternative method to standard RT-PCR or virus isolation. The assay comprised four different serotype-specific antibody pairs to detect NS1 and simultaneously identify dengue serotypes in patient sera. Serotyping performance of this original assay was fully concordant with RT-PCR results for DENV1, DENV3 and DENV4, but not for DENV2. Moreover, its overall sensitivity was still moderate (76.5%) [32]

In this study, we modified the serotyping-NS1-ELISA in order to improve DENV2 identification and to enhance overall sensitivity. The modified assay was evaluated using statistical numbers of retrospective dengue specimens infected by each of four dengue serotypes and other febrile illness (OFI) from our dengue hospital cohorts in Thailand. The results were compared with those from standard RT-PCR and the NS1-RDT kit. The serotypes identified by our modified serotyping-NS1-ELISA were concordant with the standard RT-PCR assays and overall sensitivity was increased.

## Methods

### Ethics statement

This study was approved by the ethics committee of Siriraj Institutional Review Board, Faculty of Medicine Siriraj Hospital, Mahidol University, Thailand. The written formal consent was obtained from parents or guardians before enrollment of each patient.

### Preparation of monoclonal antibodies to DENV NS1 protein (anti-NS1 Mabs) and DENV NS1 antigens

Mabs to DENV NS1 protein generated from 20 mouse hybridoma clones described previously [32,36] were used in this study. The characteristics and properties of these anti-NS1 Mabs are summarized in S1 Table. Hybridoma cells were grown and cultured in serum-free media to produce anti-NS1 Mabs, which were subsequently purified on a Protein-G HP affinity column (GE Healthcare, Uppsala, Sweden) for IgG isotypes or an IgM affinity column (GE Healthcare, Uppsala, Sweden) for IgM. The purified Mabs were biotinylated according to instruction protocols (Thermo Fisher Scientific, IL, USA) for use as detection antibodies. Soluble NS1 antigens of four DENV serotypes were obtained from culture supernatants of Vero cells infected with DENV1 (strain Hawaii), DENV2 (strain 16681), DENV3 (strain H87), DENV4 (strain H241). Soluble NS1 was purified on an immuno-affinity column coupled to anti-NS1 Mab as described previously [32] and used as standard NS1 proteins in the assay.

### Selection of highly reactive antibody pairs for each serotype by NS1 capture ELISA

Anti-NS1 Mabs were analyzed pairwise in a checkerboard manner to obtain the most reactive capture-detection antibody pairs for each serotype. Each of 20 anti-NS1 Mabs at a concentration of 10 μg/mL, as a non-labeled capture antibody (100 μL), was coated on microtiter plate wells at 4°C overnight. After blocking for 2 h with 4% Bovine serum albumin (BSA) in phosphate buffered saline (PBS), purified NS1 antigens (50 ng/mL, 100 μL) were added and incubated at 37°C for 1 h. Then, biotinylated anti-NS1 Mabs (detection antibody, marked with asterisk (*) in this study) at a concentration of 10 μg/mL (100 μL) were individually added to detect the captured NS1 antigen in each well and incubated at 37°C for 1 h. The bound biotinylated antibodies were further detected by streptavidin-HRP (1:5000) (Sigma Aldrich, IL, USA) for 1 h at 37°C. Between each step, the plates were washed four times with PBST (PBS+0.1%Tween). After adding the tetramethylbenzidine (TMB) substrate (Thermo Fisher Scientific, IL, USA), the colorimetric reaction products were measured at 450/620 nm in an ELISA plate reader (Biochrom Anthos 2010, Cambridge, UK).

### Determination of serotype-specificity of selected antibody pairs by a serotyping-NS1-ELISA

For each selected antibody pair, four ELISA wells were coated with capture antibody at 10 μg/mL in 100 μL (1 μg/well). After blocking with 4% BSA-PBS, the purified NS1 of each DENV serotype (5 ng/well) was added to each of four pre-coated wells and incubated for 1 h at 37°C. The biotinylated detection antibody (1 μg/well) was added to the four wells and incubated for 1 h at 37°C, followed with HRP-conjugated Streptavidin and TMB substrate as described above. Serotype-specific antibody pairs were chosen from the antibody pairs that showed the highest OD readings (over 1.5) to only one of four DENV serotypes.

### Determination of detection limit and cut-off values of the stNS1-ELISA

The optimal concentration of each antibody pair was analyzed to obtain high reactivity corresponding to serotype of the purified NS1 protein. The cut-off value of the assay was obtained by taking the mean OD readings of negative control antigens (mock culture supernatant or 4% BSA-PBS) plus 2 standard deviations (SD). Detection limit of the assay for each serotype was determined by the lowest NS1 concentration that gave an OD reading above the cut-off value.

### Identification of DENV serotypes of tested specimens by a modified stNS1-ELISA

Each tested plasma sample (at 1:5 dilution) was added to four wells pre-coated with the capture antibody (2E11). Four biotinylated anti-NS1 Mabs were added separately to each well to detect the captured NS1 proteins. In some cases, an additional pair of antibodies (2E11/5F3* for the DENV1/DENV3 sub-complex) was also applied to increase the sensitivity of the assay, especially for DENV1 and DENV3. In each ELISA plate, a mixture of four purified NS1 antigens (as positive control antigen) and pooled plasma from healthy individuals (as negative control) were included. NS1 was considered positive for clinical specimens when its OD reading was greater than twice the OD of the negative plasma control in each plate. The DENV serotype was identified according to the serotype-specific antibody pair that gave the highest OD reading among all four pairs tested. For the cases which were positive by none of four serotype-specific pairs, but by the additional 2E11/5F3* antibody pair, were identified as DENV1 or DENV3.

### DENV serotyping by RT-PCR

DENV serotyping was performed according to the single-tube multiplex RT-PCR previously described [37]. Briefly, isolated DENV RNA samples from patient specimens were reverse-transcribed to generate first-strand cDNA, which was subsequently amplified by PCR using pan-dengue outer primers and four pairs of serotype-specific inner primers. DENV serotype identification was based on different molecular sizes of RT-PCR products separated by agarose gel electrophoresis compared with DENV 1, 2, 3 and 4 standards (506, 346, 196 and 143 bp, respectively).

### IgM/IgG capture ELISA

The level of dengue IgM and IgG antibodies were performed by IgM/IgG capture ELISA [38]. Briefly, dengue IgM and IgG antibodies in paired plasma of acute (admission date) and convalescent (2-week follow up) phases were assayed in 96-well microplates. Anti-human IgM or IgG was coated on separate wells to capture IgM or IgG antibodies from individual specimens (diluted 1:100). Culture supernatants containing a mixture of DENV1-4 infected cells were added to the captured anti-DENV specific antibodies. The anti-E Mab (clone 4G2) was added to detect DENV-antibody complexes followed by anti-mouse IgG-HRP. After addition of TMB substrate, the OD reading was measured by spectrophotometry and IgM or IgG units were calculated. Secondary dengue infection was defined as a dengue-specific IgM/IgG ratio < 1.8, by IgM and IgG capture ELISA [39–41].

### Dengue NS1 rapid test (NS1-RDT)

The SD BIOLINE Dengue NS1 Ag Rapid Test (Abbot, FL, USA) was used as a representative commercial NS1-RDT kit in this study. Assays were performed according to the manufacturer’s instructions. Briefly, each tested plasma sample (100 μL) was added to a sample well on the cassette to allow plasma migration for 15–30 min. Positive cases were interpreted by the appearance of both test and control lines. In contrast, the reactivity of the control line alone indicated a negative result, but confirmed the quality of the devices.

### Clinical specimens and laboratory dengue diagnosis

A panel of acute dengue clinical specimens was selected from our dengue clinical cohorts of enrolled pediatric patients (aged 2–14 years) who were admitted to Khon Kaen and Songkhla hospitals, Thailand, during 2002 to 2012. All patient inclusion and blood sampling occurred after obtaining written informed consent from the patient’s parents or guardians. The patients were clinically diagnosed as DF/DHF according to WHO criteria (1997) [42]. Secondary dengue infection was defined as a dengue-specific IgM/IgG ratio < 1.8, [39–41]. DENV infection was confirmed for all patients by either of two laboratory diagnoses including viral RNA detection by RT-PCR [37] and IgM/IgG seroconversion. Infecting DENV serotype of each patient was identified by RT-PCR with serotype-specific primers [37]. Patients who were negative by both laboratory assays were identified as other febrile illness (OFI) cases. A panel of specimens comprised 250 samples in total including 200 RT-PCR confirmed dengue infection and 50 OFI cases were used in this study. DENV-infected specimens were re-analyzed with RT-PCR to confirm their serotypes prior to be used. The final numbers of specimens for each serotype were 50, 55, 45 and 50 for DENV1 to DENV4, respectively (S1 Fig). The specimens were obtained during the febrile phase (1–4 day of fever, before defervescence date). Most of DENV-infected samples were identified as secondary infection (98%). The numbers of DF/DHF patients were 78/121. Immune status and disease severity of acute-phase clinical specimens in this study are summarized in S2 Table.

### Statistical analysis

Samples size was calculated based on expected sensitivity and specificity at 85% with an acceptable margin of error of ±10% [43]. The confident level was set at 95% (i.e. alpha = 0.05) (about 49 specimens were required for each assessment group). An agreement between stNS1-ELISA (plus) and NS1-RDT was assessed by Cohen’s kappa and interpreted based on previous recommendation [44]. McNemar’s exact tests were used to compare the performance of stNS1-ELISA (plus) and NS1-RDT. All 95% confidence intervals were computed by inverting the score of one-sample proportions tests without continuity correction. All statistical analyses were performed with R programming language (version 3.6.1, R Foundation for Statistical Computing, Vienna, Austria).

### Competitive binding ELISA

Purified DENV1-4 NS1 proteins from Vero cells infection were coated in ELISA wells at 50 ng/well in a coating buffer (0.1 M Carbonate-Bicarbonate Buffer pH 9.2) at 4°C overnight incubation. The coated wells were washed four times with 0.1% Tween 20 in PBS (pH 7.4) and blocked with 4%BSA in PBS for 1 h at 37°C. The non-labelled antibody as a blocking antibody 50 μg/ml 100 μl (5 μg/well) was firstly added to the wells and incubated for 1 h at 37°C. The biotin-labeled antibody was later added into wells for the final concentration of 6 μg/ml and were incubated for another 1 h at 37°C to bind the available epitopes. The immune complexes were detected with Streptavidin-HRP for 1 h, followed with TMB substrate (Thermo Fisher Scientific, USA) and measured OD reading by ELISA reader at A450/620 nm.

The percentage of blocking was calculated by the difference between OD reading of control wells with no blocking antibody and those with blocking antibody, then divided by OD reading of control wells x 100. The physical distance between spatial binding epitopes of two antibodies is anti-correlated with % blocking. (> 70%blocking, closely or overlapped epitope; 20–70% blocking, partially overlapped epitope; <20% blocking, discrete or non-overlapped epitope).

### Surface Plasmon Resonance (SPR)

Affinity and Kinetic binding between NS1 proteins and anti-NS1 antibodies were analyzed by Surface Plasmon Resonance technique using Biacore X100 (GE Healthcare, USA). Briefly, recombinant NS1 protein (500 RU) was captured using Sensor Chip CM5 (GE Healthcare, USA). Then, anti-NS1 mAbs at 50 nM was flown over the chip surface through running buffer (1xHBS-N and 0.005% P20). Antibody was injected for 360 s and disassociated for 720 s. The sensorgram and affinity constant (K_D_) were calculated by Biacore X100 software. Three replications were performed from each DENV serotype.

## Results

### Selection of new antibody pairs to detect DENV NS1 of each serotype in the modified stNS1-ELISA

**Fig 1:**
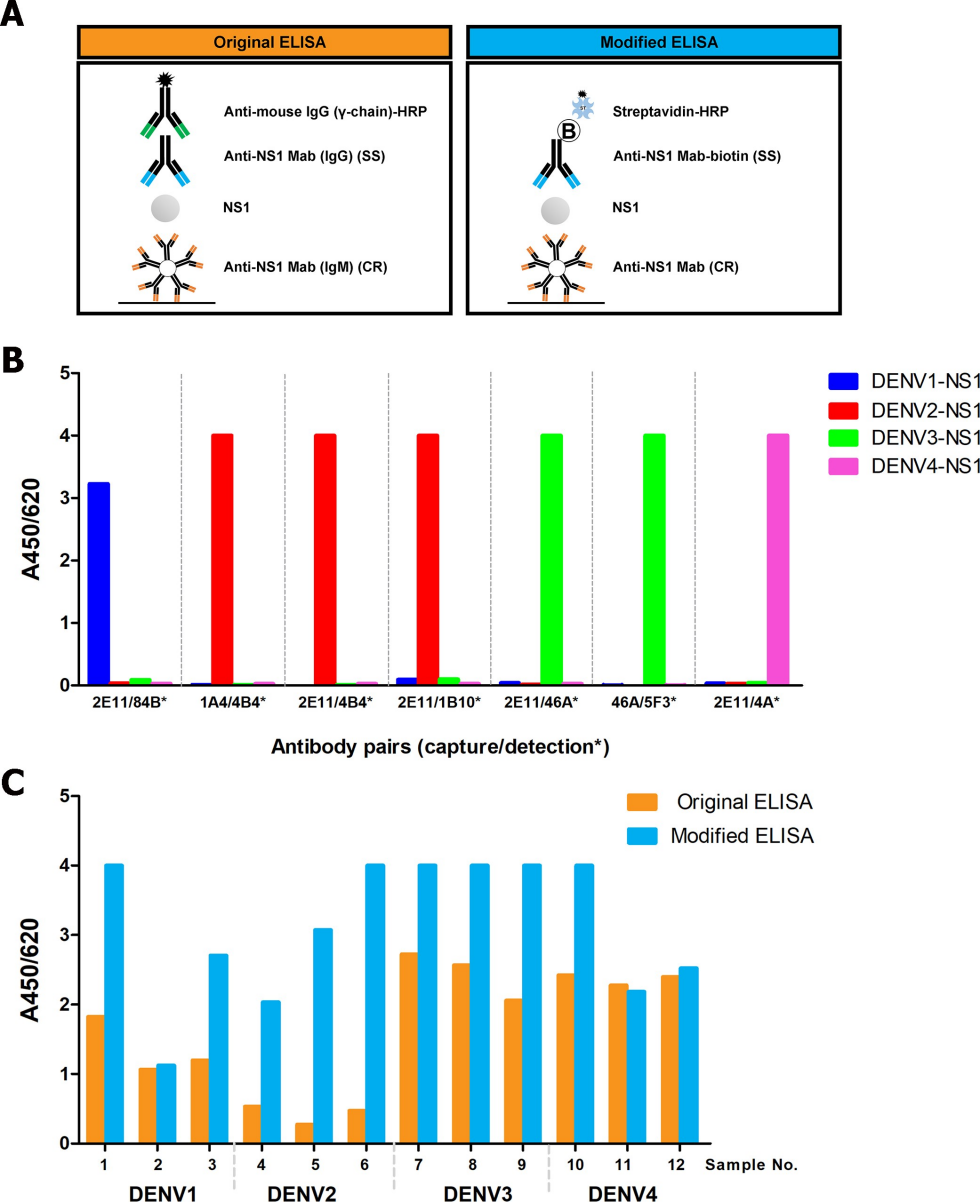
Selection of new antibody pairs to NS1 protein of four DENV serotypes by the modified stNS1-ELISA. A) Schematic diagram of stNS1-ELISA in the original format (left) and the modified format (right); CR = cross reactive, SS = serotype-specific. B) Serotype-specificity of 8 selected antibody pairs to NS1 from each DENV serotype. Each antibody pair is composed of capture MAb and biotinylated detection MAb (*). DENV1 NS1 (blue), DENV2 NS1 (red), DENV3 NS1 (green), DENV4 NS1 (pink). C) Comparison of OD readings of clinical samples obtained from the original format (orange) and the modified format (blue).

In order to improve sensitivity and serotype identification of stNS1-ELISA, we sought new antibody pairs from our 20 distinct clones producing anti-NS1 Mabs (S1 Table). The limitation of the original stNS1–ELISA (Fig 1A, left) was due to the use of non-labeled antibodies, therefore the capture and detection Mabs in each antibody pair were restricted to those that have different immunoglobulin isotypes. In this study, the assay was modified by biotinylation of all detection Mabs to enable pairing with capture Mabs of any isotype (Fig 1A, right) and to enhance their reactivity using the streptavidin-HRP detection system. The capture Mabs and biotinylated detection Mabs (marked as *) were analyzed pairwise in a checkerboard manner by NS1 capture ELISA for each serotype. Eight antibody pairs which provided strong reactivity to a DENV NS1 serotype with no cross reactivity to NS1 of other serotypes were selected namely: DENV1, 2E11/84B*; DENV2, 1A4/4B4*, 2E11/4B4*, 2E11/1B10*; DENV3, 2E11/46A*, 46A/5F3*; DENV4, 2E11/4A*, 4A/S9.6* (Fig 1B). It should be noted that three pairs, i.e. 2E11/84B*, 2E11/46A* and 2E11/4A* were the same antibody pairs as used in the original stNS1-ELISA for DENV1, DENV3 and DENV4, respectively.

These antibodies were also analyzed for relative binding epitopes on NS1 of each serotypes by competitive binding ELISA. We found that each antibody did not block NS1 binding of other antibodies in the same serotype, while self-blocking of antibodies were shown. (S3 Fig). The results demonstrated that these antibodies bind to discrete epitopes on DENV NS1.

Eight selected antibody pairs were firstly pre-validated in a small numbers of patient specimens. Nineteen samples were randomly selected from a panel of 250 specimens which included all four DENV serotypes (DENV1, n = 4; DENV2, n = 4; DENV3, n = 3; DENV4, n = 4) and OFI (n = 4), and tested by stNS1-ELISA. The specimens were tested side by side in the modified format with four antibody pairs in the original format (Table 1). Equivalent numbers of NS1 positive cases between both ELISA formats were found for DENV1 (2E11/84B*) and DENV4 (2E11/4A*). One fewer DENV4 positive case was found for 4A/S9.6* compared with 2E11/4A*; therefore this antibody pair was excluded. All three DENV3 specimens were detected in the modified assay with 2E11/46A* and 46A/5F3*, but the antibody pair 2E11/46A* was preferable to the other as it shared the same capture antibody (2E11) with other serotypes, thus minimizing the assay costs. Among the three candidate antibody pairs for DENV2 detection, 2E11/1B10* detected all four tested samples, while another two (1A4/4B4*, 2E11/4B4*) and the original pair (2E11/3D1) could detect only one sample. The 2E11/1B10* antibody pair was therefore chosen as the DENV2-specific detection pair. Of note, the OD readings obtained from the modified stNS1-ELISA were markedly higher than those from the original format for the same specimens (Fig 1C), suggesting the enhancement of signal reactivity in the biotinylated modified format.

**Table 1:** Preliminary testing of the modified stNS1 ELISA compared with the original format in clinical samples.

Clinical samples	Sample No.	RT-PCR results	stNS1 ELISA												
			Original ELISA				Modified ELISA								
			D1	D2	D3	D4	D1	D2	D2	D2	D3	D3	D4	D4	Dengue serotype
			2E11	2E11	2E11	2E11	2E11	1A4	2E11	2E11	2E11	46A	2E11	4A	Capture Mab
			84B	3D1	46A	4A	84B*	4B4*	4B4*	1B10*	46A*	5F3*	4A*	S9.6*	Detection Mab (*)
DENV infection	**1**	DENV1	**+**	-	-	-	**+**	-	-	-	-	-	-	-	
	**2**	DENV1	**+**	-	-	-	**+**	-	-	-	-	-	-	-	
	**3**	DENV1	**+**	-	-	-	**+**	-	-	-	-	-	-	-	
	**4**	DENV1	**+**	-	-	-	**+**	-	-	-	-	-	-	-	
	**5**	DENV2	-	-	-	-	-	-	-	**+**	-	-	-	-	
	**6**	DENV2	-	-	-	-	-	-	-	**+**	-	-	-	-	
	**7**	DENV2	-	+	-	-	-	+	+	**+**	-	-	-	-	
	**8**	DENV2	-	-	-	-	-	-	-	**+**	-	-	-	-	
	**9**	DENV3	-	-	**+**	-	-	-	-	-	**+**	**+**	-	-	
	**10**	DENV3	-	-	**+**	-	-	-	-	-	**+**	**+**	-	-	
	**11**	DENV3	-	-	**+**	-	-	-	-	-	**+**	**+**	-	-	
	**12**	DENV4	-	-	**-**	**+**	-	-	-	-	-	-	**+**	**+**	
	**13**	DENV4	-	-	**-**	**+**	-	-	-	-	-	-	**+**	-	
	**14**	DENV4	-	-	-	-	-	-	-	-	-	-	-	-	
	**15**	DENV4	-	-	**-**	**+**	-	-	-	-	-	-	**+**	**+**	
OFI	**16**	Neg	-	-	-	-	-	-	-	-	-	-	-	-	
	**17**	Neg	-	-	-	-	-	-	-	-	-	-	-	-	
	**18**	Neg	-	-	-	-	-	-	-	-	-	-	-	-	
	**19**	Neg	-	-	-	-	-	-	-	-	-	-	-	-	

As a result, four antibody pairs were selected for the modified stNS1-ELISA (DENV1, 2E11/84B*; DENV2, 2E11/1B10*; DENV3, 2E11/46A* and DENV4, 2E11/4A*), in which biotinylated serotype-specific antibodies were used as detection antibodies paired with a flavivirus cross-reactive capture antibody shared among all four serotypes. Among the four antibody pairs, only DENV2, 2E11/1B10* was new, while others were the same clones as used in the original assay. The detection limit of each antibody pair to purified NS1 for DENV1 to DENV4 was below 1 ng/mL, lower than that of the original format (Table 2). Additionally, the assay is specific to only NS1 of homologous DENV serotypes, as no cross-reactivity was observed with other serotypes or other flaviviruses such as Zika and Japanese encephalitis virus (JEV). (S2 Fig).

**Table 2:** Comparison of the antibody pairs and detection limit between the original and the modified stNS1-ELISA.

Dengue serotypes	Original ELISA		Modified ELISA	
	Antibody pairs (Capture Ab/ detection Ab)	Detection limit (ng/mL)^#^	Antibody pairs (Capture Ab/ detection Ab*)	Detection limit (ng/mL)^#^
DENV1	2E11/84B	4.4	2E11/84B*	0.20
DENV2	2E11/3D1	15.8	2E11/1B10*	0.02
DENV3	2E11/46A	2.9	2E11/46A*	0.02
DENV4	2E11/4A	1.7	2E11/4A*	0.05

* Biotinylated antibody.

# Obtained from the lowest NS1 concentration which gave the OD reading above the cut-off value (mean OD reading of negative control antigen (4% BSA-PBS or mock culture sup) + 2 standard deviations (SD).

### Evaluation of the modified stNS1-ELISA with clinical specimens

The modified stNS1-ELISA was fully evaluated with a panel of 250 clinical samples, consisting of 200 acute-phase plasma samples from confirmed dengue patients which were RT-PCR positive (DENV1, n = 50; DENV2, n = 45; DENV3, n = 55; DENV4, n = 50) and 50 acute-phase plasma samples from OFI cases. Among 200 dengue cases, 156 were positive for one of the four antibody pairs, suggesting 78.0% overall sensitivity, slightly increased from 76.5% as previously reported in the original ELISA [32]. However, prediction of DENV serotypes based on NS1 positive samples were in 100% concordance with the standard RT-PCR method for all four serotypes. The results suggested an improvement of DENV2 serotype identification compared with the original stNS1-ELISA (100% vs 82.4%). All OFI clinical samples were negative, indicating 100% specificity of the modified stNS1–ELISA (Table 3). The sensitivity to DENV2 (83.6%), DENV3 (84.4%) and DENV4 (82.0%) infected cases was higher than the 75–80% sensitivity in the original ELISA to detect these serotypes. Unexpectedly, lower sensitivity was found for DENV1 (62%) vs 74% in the original ELISA although the same antibody pair was used, suggesting the performance of this DENV1 antibody pair in clinical samples is varied.

**Table 3:** Performance evaluation of the modified stNS1-ELISA vs. RT-PCR in clinical specimens.

Clinical samples diagnosed by RT-PCR	Modified stNS1-ELISA					
	Numbers of positive samples					Numbers of negative samples
	2E11/84B* DENV1	2E11/1B10* DENV2	2E11/46A* DENV3	2E11/4A* DENV4	Total	
DENV1 (n = 50)	31	0	0	0	31	19
DENV2 (n = 55)	0	46	0	0	46	9
DENV3 (n = 45)	0	0	38	0	38	7
DENV4 (n = 50)	0	0	0	41	41	9
Total (n = 200)	**31**	**46**	**38**	**41**	156	44
OFI (n = 50)	0	0	0	0	0	50
Grand total (n = 250)	31	46	38	41	156	94
% Serotype agreement to PCR	100% (31/31)	100% (46/46)	100% (38/38)	100% (41/41)		
% Sensitivity to each serotype	62.0% (31/50)	83.6% (46/55)	84.4% (38/45)	82.0% (41/50)		
% Overall sensitivity	78% (156/200)					
% Overall specificity	100% (50/50)					
% Accuracy for dengue diagnosis	82.4% (206/250)					

### Improvement of DENV1 performance in the modified stNS1-ELISA by an additional antibody pair 2E11/5F3*

**Fig 2:**
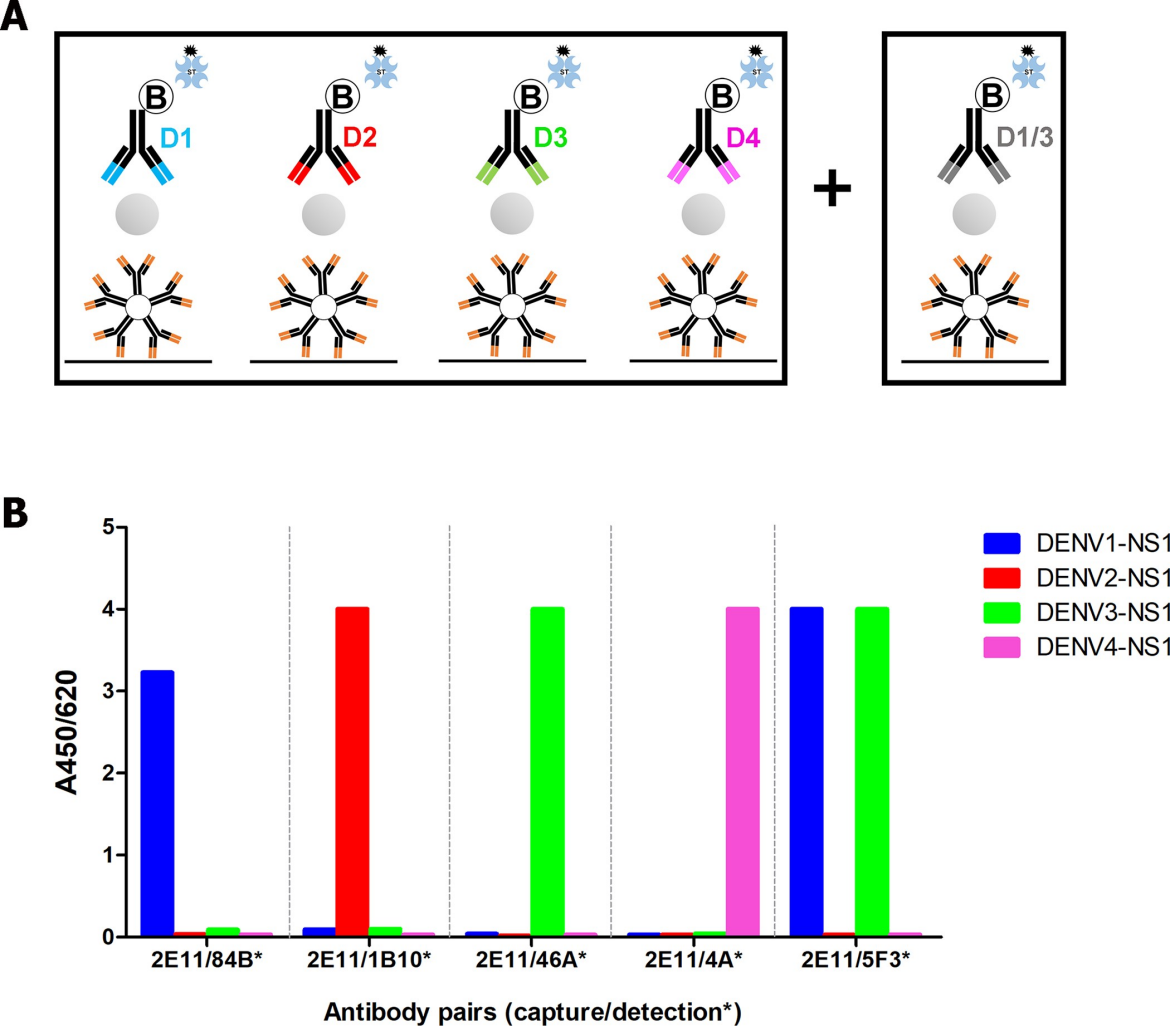
Modified stNS1-ELISA (plus). (A) Schematic diagram of the modified stNS1-ELISA (plus) containing five antibody pairs, four of which were serotype-specific and the other was DENV1/3 sub-complex. (B) Reactivity of antibody pairs to purified NS1 of each dengue serotype.

Based on our checkerboard screening results, 2E11/84B* was the only suitable antibody pair for DENV1 serotype-specific detection. The 2E11/5F3* pair showed better reactivity with DENV1 NS1 in comparison with 2E11/84B*, while it was also cross reactive with DENV3 NS1, but not with DENV2 NS1 or DENV4 NS1 (Fig 2). Relative binding epitope of both 5F3 and 84B on DENV1 NS1 as well as that of 5F3 and 46A on DENV3 NS1 were analyzed by a competitive binding ELISA (S3 Fig). The results showed that no block binding between 5F3 and 84B or 5F3 and 46A were observed, suggesting that 5F3 recognized non-overlapping or discrete epitopes to 84B or 46A. In addition, the binding affinity of 5F3 and 84B to DENV1 NS1 was also investigated by Surface Plasmon Resonance. We found that the equilibrium dissociation constant (K_D_) of 5F3 (4.72 nM) was lower than that of 84B (54.8 nM) (S3 Table), suggesting a much stronger binding affinity of 5F3 over 84B to DENV1 NS1. Similar result was found with 5F3 and 46A to DENV3 NS1 in that 5F3 showed stronger binding affinity over 46A (K_D_ of 5F3 and 46A were 2.44 and 12.3 nM, respectively) (S3 Table).

We therefore tested 2E11/5F3* in 26 samples which were negative for DENV1 (2E11/84B*, n = 19) and DENV3 (2E11/46A*, n = 7) in the modified stNS1-ELISA. Those that were negative for DENV2 or DENV4, which were non-binding serotypes to 2E11/5F3*, were not tested. Interestingly, 15 of 26 cases (57.7%) were positive with 2E11/5F3*, 14 of which (93.3%) were assigned as DENV1 cases by RT-PCR and the other was assigned as DENV3 (Table 4). This result suggested that 2E11/5F3* could improve overall sensitivity of the modified stNS1-ELISA, in particular for detection of DENV1 cases in this panel of clinical specimens. The modified stNS1-ELISA with additional antibody pair 2E11/5F3* is further designated as “the modified stNS1-ELISA (plus)”. With this method, the sensitivity to serotype can be increased up to 90.0% (45/50) for DENV1 and 86.7% (39/45) for DENV3 and overall sensitivity increased to 85.5% (171/200) (Table 4).

**Table 4:** Performance evaluation of the modified stNS1-ELISA (plus) vs. RT-PCR in clinical specimens.

Clinical samples diagnosed by RT-PCR	Modified stNS1-ELISA (plus)						
	Numbers of positive samples						Numbers of negative samples
	2E11/84B* DENV1	2E11/1B10* DENV2	2E11/46A* DENV3	2E11/4A* DENV4	2E11/5F3* DENV1/3	Total	
DENV1 (n = 50)	31	0	0	0	14	45	5
DENV2 (n = 55)	0	46	0	0	ND^#^	46	9
DENV3 (n = 45)	0	0	38	0	1	39	6
DENV4 (n = 50)	0	0	0	41	ND^#^	41	9
Total (n = 200)	**31**	**46**	**38**	**41**	15	171	29
OFI (n = 50)	0	0	0	0	ND^#^	0	50
Grand total (n = 250)	31	46	38	41	15	171	79
% Serotype agreement to PCR	100% (31/31)	100% (46/46)	100% (38/38)	100% (41/41)			
% Sensitivity to each serotype*	**90.0% (45/50)**	83.6% (46/55)	**86.7% (39/45)**	82.0% (41/50)			
% Overall sensitivity	85.5% (171/200)						
% Overall specificity	100% (50/50)						
% Accuracy for dengue diagnosis	88.4% (221/250)						

ND^#^ = not done.

### Comparison of the modified stNS1-ELISA (plus) performance with a commercial dengue NS1-RDT

The assay performance of our modified stNS1-ELISA (plus) was compared with a commercial dengue NS1-RDT by using the same panel of clinical specimens. The dengue NS1-RDT from Standard Diagnostic was selected for comparison as it is considered one of the best kits currently available [28,45]. In comparison with the standard RT-PCR method, only 147 of 200 confirmed DENV cases were positive by NS1-RDT, with an overall sensitivity of 73.5%. Regarding dengue serotypes, the NS1-RDT was the most sensitive for DENV1 (80.0%), followed by DENV3 (77.8%), DENV2 (69.1%) and DENV4 (68.0%). One false positive case was found among the 50 OFI samples tested with NS1-RDT, leading to specificity of 98.0% (Table 5), but it is not significantly different to the stNS1–ELISA (Table 6). The agreement between stNS1-ELISA (plus) and NS1-RDT was substantial with Cohen’s kappa 0.79 (95%CI 0.71–0.86). The performances of the modified stNS1-ELISA (plus) was compared with that of NS1–RDT (Table 6). The results indicated that the modified stNS1-ELISA (plus) showed significantly superior performance to NS1-RDT (85.5% vs 73.5%, *P* <0.001). Regarding the sensitivity to detect each DENV serotype, the modified stNS1-ELISA (plus) showed significantly greater sensitivity than NS1-RDT for DENV2 and DENV4. but not significant for DENV3. The modified stNS1-ELISA (plus) assay with addition of 2E11/5F3* had the sensitivity for DENV1 detection surpassing that for NS1-RDT albeit not significant (Table 6).

**Table 5:** Performance of dengue NS1-RDT vs. standard RT-PCR.

NS1-RDT	RT-PCR confirmed acute Dengue samples					Non-dengue samples or OFI	Total
	DENV1	DENV2	DENV3	DENV4	Total		
**Positive (+)**	40	38	35	34	147	1	148
**Negative (-)**	10	17	10	16	53	49	102
**Total**	50	55	45	50	200	50	250
**% sensitivity to serotype**	80.0% (40/50)	69.1% (38/55)	77.8% (35/45)	68.0% (34/50)			
**% sensitivity**	73.5% (147/200)						
**% specificity**	98.0% (49/50)						

**Table 6:** Comparison of overall performance of the modified stNS1-ELISA, the modified stNS1-ELISA (plus) and NS1-RDT.

Assays	% overall sensitivity [95% CI]	% overall Specificity [95% CI]	% Diagnostic accuracy [95% CI]	% Sensitivity to serotype [95% CI]			
				DENV1	DENV2	DENV3	DENV4
1. Modified stNS1-ELISA (4-pairs)	78.0 (156/200) [71.8–83.2]	100 (50/50) [92.9–100.0]	82.4 (206/250) [77.2–86.6]	62.0 (31/50) [48.2–74.1]	83.6 (46/55) [71.7–91.1]	84.4 (38/45) [71. 2–92.3]	82.0 (41/50) [69.2–90.2]
2. Modified stNS1-ELISA(plus) (5-pairs)	85.5 (171/200) [80.0–89.7]	100 (50/50) [92.9–100.0]	88.4 (221/250) [83.8–91.8]	90.0 (45/50) [78.6–95.7]	83.6(46/55) [71.7–91.1]	86.7 (39/45) [73.8–93.7]	82.0 (41/50) [69.2–90.2]
3. SD-NS1 RDT	73.5 (147/200) [70.0–79.1]	98.0 (49/50) [89.5–99.6]	78.4 (196/250) [73.0–83.1]	80.0 (40/50) [67.0–88.8]	69.1 (38/55) [56.0–79.7]	77.8 (35/45) [63.7–87.5]	68.0 (34/50) [54.2–79.2]
Exact McNemar test *P*-value (2 versus 3)	<0.001*	1.000	<0.001*	0.063	0.008*	0.125	0.016*

(*) indicated significantly difference between two assays (P < 0.05).

## Discussion

We previously developed the original stNS1-ELISA to distinguish four DENV serotypes as an alternative method to standard RT-PCR or virus isolation. The assay comprised four different serotype-specific antibody pairs to detect NS1 and simultaneously identify DENV serotypes in patient sera. Serotyping performance of this original assay was fully concordant with RT-PCR results for DENV1, DENV3 and DENV4, but not for DENV2. Moreover, its overall sensitivity was still moderate (76.5%) [32]. To increase overall sensitivity, in particular for the DENV2 serotype, the stNS1-ELISA has been modified in this study. Instead of using a different immunoglobulin isotype for each antibody pair (IgM capture and IgG detection antibody) as in the original platform, the detection antibody was labeled with biotin and detected with streptavidin-HRP. This modified format not only enhanced signal strength, but also gave us the opportunity to match new antibody pairs without isotype concern for all serotypes. Nevertheless, the same antibody pairs from the original format for DENV1, DENV3 and DENV4 serotypes were confirmed as the best serotype-specific antibody pairs among our available antibody panel. The new antibody pair 2E11/1B10* was selected for DENV2 detection over two other potential DENV2-specific pairs (1A4/4B4* and 2E11/4B4*) due to its superior performance in initial testing of patient specimens (Table 1). We demonstrated that 1B10 did not block DENV2 NS1 binding to others by competitive binding ELISA (S3 Fig), suggesting that it recognized a distinct epitope from others. Kinetic binding affinities of these antibodies to DENV2 NS1 as analyzed by Surface Plasmon Resonance (SPR) showed that their equilibrium dissociation constants (K_D_) were not much different (S3 Table). Therefore, the high performance of 1B10 may be due to a greater exposure of the 1B10 epitope on the surface of NS1 molecules, which would be more easily accessible than others. In a previous study, 1B10 was neglected in the original ELISA as it showed cross reactivity to DENV1, 2 and 3 by NS1 capture ELISA and FACS analysis, although it was DENV2-specific by dot blot and western blot analysis [32]. However, by several rounds of limiting dilution to achieve single cell cloning of 1B10, we finally obtained DENV2-specific behavior of 1B10 by stNS1-ELISA and thus included it in this study. Currently epitope mapping of 1B10 is under investigation.

In this modified ELISA, we found that sensitivity to the DENV1 serotype was less than that of the original format (62% vs 73.9%) [32], although the same antibody pair (2E11/84B*) was used with an enhanced biotinylation detection system. Besides the different sets of tested DENV1 specimens, we postulated that 84B may recognize a highly conformational epitope on DENV1 NS1 where it is easily hindered and varied among individuals. However, the addition of 5F3 could improve the sensitivity of DENV1 NS1 (from 62% to 90). Additionally, we observed that OD readings of the positive samples were markedly higher with 2E11/5F3* than with 2E11/84B*. This result suggested that the 5F3 epitope may be more accessible than that of 84B. We also showed that both 5F3 and 84B recognized non-overlapping epitopes on DENV1 NS1 by a competitive binding ELISA (S3 Fig). Binding affinity of 5F3 to DENV1 NS1 or DENV3 NS1 was stronger than 84B or 46A as analyzed by SPR. (S3 Table) Nevertheless, according to clinical evaluation in this study, the additional antibody pair 2E11/5F3* seems to be more advantageous for the detection of DENV1 than DENV3. It would suggest that the degree of epitope accessibility of 5F3 and 46A to DENV3 NS1 might not differ by much, though their detailed epitopes have not yet been mapped. The replacement of new DENV1-serotype specific antibody pairs is therefore still needed to improve the sensitivity to DENV1 and to minimize the complexity of assay interpretation. Due to Flavivirus-cross-reactivity of 2E11 to other flaviviruses such as Zika and Japanese encephalitis viruses, which co-circulate in the same endemic regions [46,47], it is possible to expand the multiplex assay to distinguish DENV from these particular flaviviruses if specific NS1 antibodies to these flaviviruses are available.

The lower sensitivity of NS1 antigen assays compared to RT-PCR is caused by false negative results found in some dengue specimens. One reason is due to the presence of immune complexes between the secreted NS1 antigen and existing anti-NS1 antibodies in the blood circulation, especially in human populations living in dengue endemic areas [48–50]. These complexes may hinder epitope recognition by anti-NS1 antibodies in the NS1 assay. Dissociation of these immune complexes by various pretreatment methods (for example, heat or acid treatment) has been shown to enhance the sensitivity of DENV NS1 assays [49,51–53]. However, to our experiences, we observed that heat- or acid-pretreated specimens sometimes altered serotype-specific interpretation to be negative or cross reactive to other serotypes, possibly due to the disruption of epitopes recognized by conformation-dependent antibodies. Therefore, other milder methods to dissociate immune complexes without disturbing epitope conformation should be explored. Alternatively, new serotype-specific antibodies recognizing denatured or linear epitopes of NS1 may be more effective for testing pretreated specimens, as previously reported by Gelanew et al [54].

Clinical evaluation of NS1 assays in several studies showed that DENV4 has the lowest sensitivity among the four DENV serotypes [26,55–57], which agrees with our observation for NS1-RDT (68% sensitivity to DENV4). In contrast, for the stNS1-ELISA (plus), DENV4 is detected with comparable sensitivity to DENV2 and DENV3. The lower sensitivity for DENV1 could be enhanced by further testing with 2E11/5F3* It was agreed by several groups that sensitivity of the NS1 assays is greater in primary than secondary infection cases [13,26,28,57]. In our study, regardless of serotype prediction, stNS1-ELISA (plus) shows remarkably high sensitivity (over 80%) to NS1 of all four DENV serotypes, although most of tested specimens were from secondary infection (S2 Table). This may suggest that our NS1 assay is preferable in dengue endemic regions where secondary infected cases are frequently found.

In conclusion, we have established the modified stNS1-ELISA (plus) as a dengue serotyping method that is more sensitive than the original stNS1-ELISA. The serotypes inferred from stNS1-ELISA (plus) are in 100% agreement with the standard RT-PCR method. An additional antibody pair (2E11/5F3*) enhanced the sensitivity to DENV1, which consequently increased overall assay sensitivity. For early dengue diagnosis and serotype identification, the stNS1-ELISA has advantages over RT-PCR including, (i) plasma samples can be assayed directly without pre-processing, (such as RNA extraction and reverse transcription as required for RT-PCR), (ii) relatively low technological and resource requirements thus making it suitable for resource-poor settings, (iii) the window of diagnosis is extended due to the longer presence of NS1 antigen in blood circulation compared with the RNA genome. Taken together, the modified stNS1-ELISA (plus) is a promising high-throughput and alternative method to identify DENV serotypes in patient specimens for disease surveillance and epidemiological studies. This method can reduce complicated workloads and costs of laboratory setup needed by RT-PCR for identification of DENV serotypes, especially in limited resource settings where DENV disease is endemic.

## Supporting information

S1 Fig Schematic diagram of selected panels of dengue clinical specimens for assay validation. (PDF)

S2 Fig Reactivity of anti-NS1 antibody pairs to NS1 of DENV, but not other flaviviruses. Each antibody pair, composed of capture MAb and biotinylated detection MAb (*), were tested against NS1 antigens derived from dengue and other flaviviruses (infected culture supernatants) by the modified NS1-ELISA (plus). The existence of NS1 antigens were demonstrated by reactivity against anti-NS1 2E11 Mab by dot blot assay (right). DENV1NS1 (blue), DENV2NS1 (red), DENV3NS1 (green), DENV4NS1 (pink), ZikaNS1 (turquoise), JEVNS1 (magenta), Mock (black).
(TIF)

S3 Fig Determination of relative binding epitopes of two antibodies by competitive binding NS1 ELISA. The non-labelled antibody was added to pre-coated NS1 antigen as a blocking antibody. The biotinylated antibody (*) was later added as a detection antibody. The same clone for blocking and biotin-labeled antibody was included as a positive control for self-epitope blocking. Below 20% blocking indicated non-overlapped or discrete binding epitope of two antibodies.
(TIF)

S1 Table Characteristics and properties of anti-NS1 Mabs used in this study. (PDF)

S2 Table Summary of immune status, severity and DENV serotypes of acute-phase patients’ plasma used in this study. (PDF)

S3 Table Binding kinetics of anti-NS1 Mabs to DENV NS1 antigen by Surface Plasmon Resonance (SPR). (PDF)
