# Supplementary figures and images for: High performance dengue virus antigen-based serotyping-NS1-ELISA (plus): A simple alternative approach to identify dengue virus serotypes in acute dengue specimens
Source: PLoS Negl Trop Dis. 2021 Feb 26;15(2):e0009065. doi: 10.1371/journal.pntd.0009065 (PMC7946175; doi:10.1371/journal.pntd.0009065)

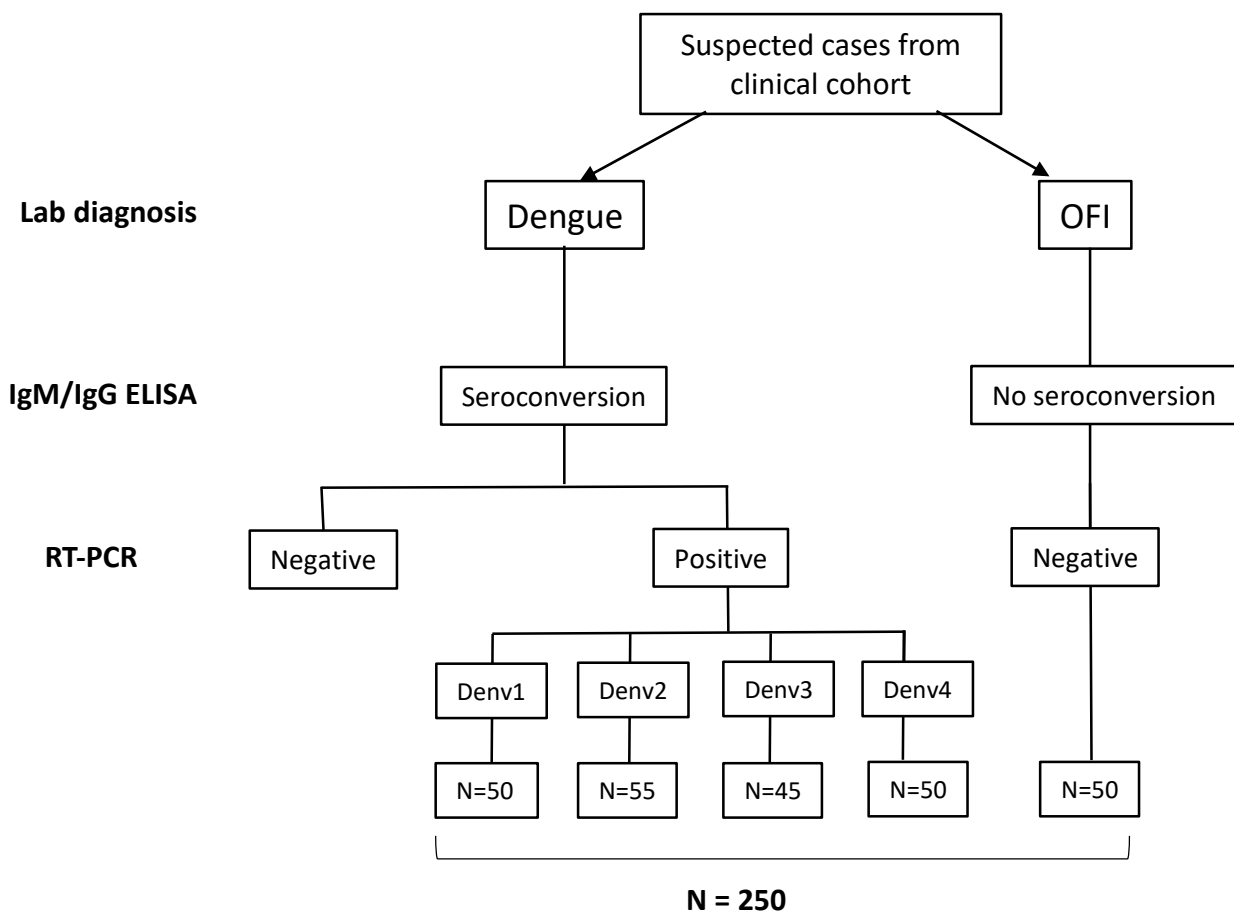

**S1 Fig. Schematic diagram of selected panels of dengue clinical specimens for assay validation.**

Supplement: S1 Fig — (PDF) [file pntd.0009065.s001.pdf]

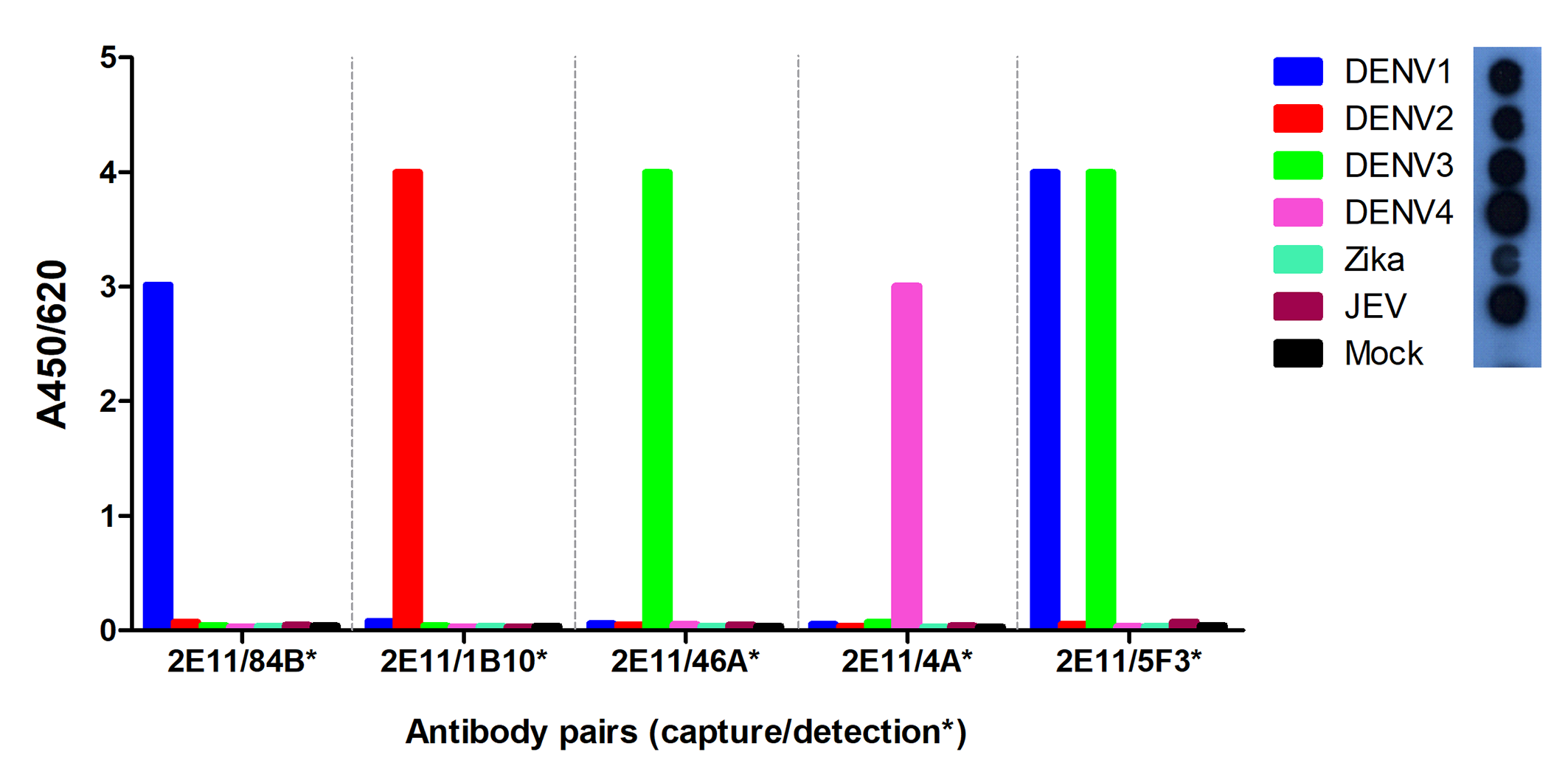

Supplement: S2 Fig — Each antibody pair, composed of capture MAb and biotinylated detection MAb (*), were tested against NS1 antigens derived from dengue and other flaviviruses (infected culture supernatants) by the modified NS1-ELISA (plus). The existence of NS1 antigens were demonstrated by reactivity against anti-NS1 2E11 Mab by dot blot assay (right). DENV1NS1 (blue), DENV2NS1 (red), DENV3NS1 (green), DENV4NS1 (pink), ZikaNS1 (turquoise), JEVNS1 (magenta), Mock (black). (TIF) [file pntd.0009065.s002.tif]

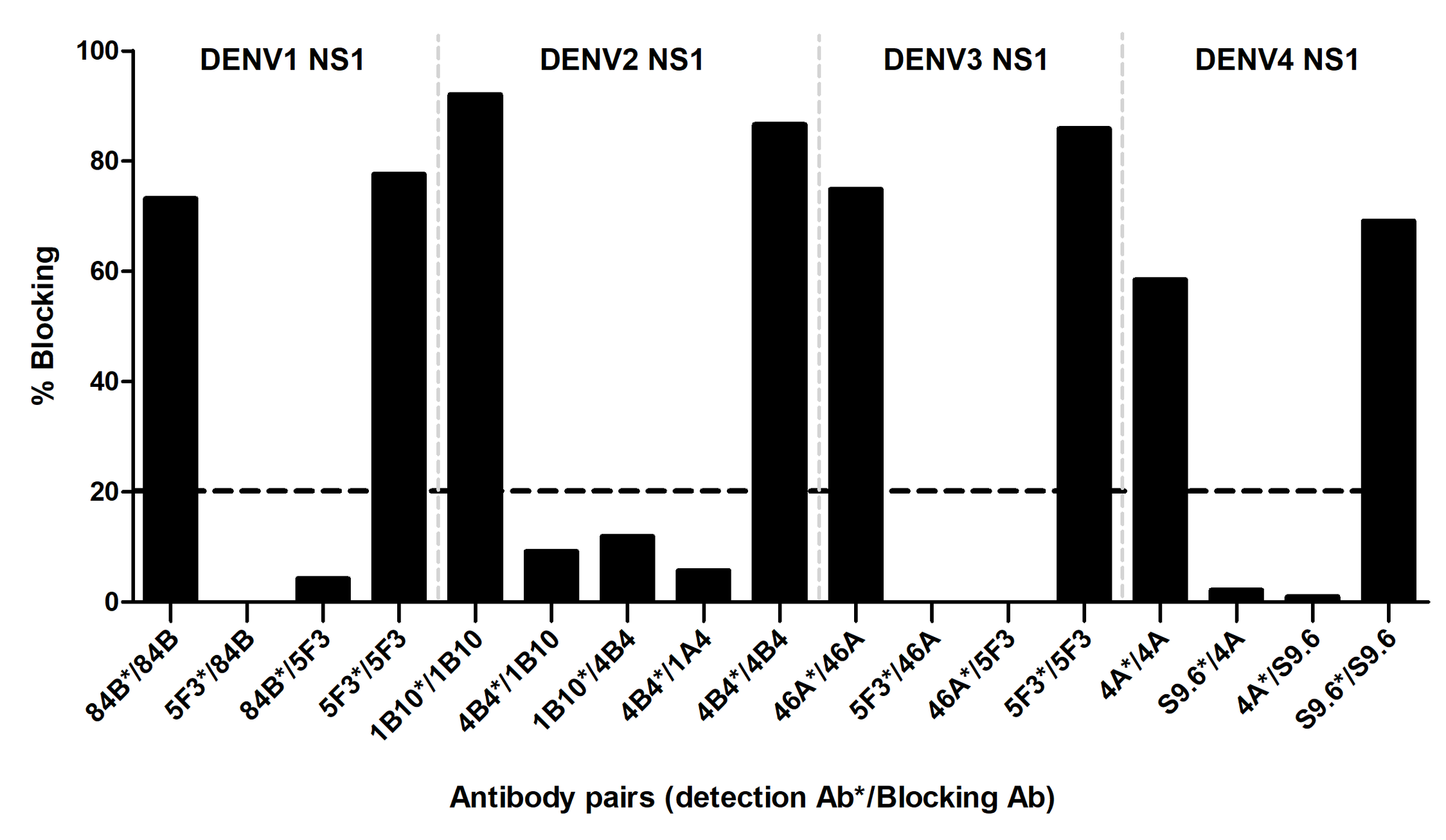

Supplement: S3 Fig — The non-labelled antibody was added to pre-coated NS1 antigen as a blocking antibody. The biotinylated antibody (*) was later added as a detection antibody. The same clone for blocking and biotin-labeled antibody was included as a positive control for self-epitope blocking. Below 20% blocking indicated non-overlapped or discrete binding epitope of two antibodies. (TIF) [file pntd.0009065.s003.tif]
