# Supplementary material for: High performance dengue virus antigen-based serotyping-NS1-ELISA (plus): A simple alternative approach to identify dengue virus serotypes in acute dengue specimens
Source: PLoS Negl Trop Dis. 2021 Feb 26;15(2):e0009065. doi: 10.1371/journal.pntd.0009065 (PMC7946175; doi:10.1371/journal.pntd.0009065)
Supplement: S1 Table — (PDF) [file pntd.0009065.s004.pdf]

S1 Table. Characteristics and properties of anti-NS1 Mabs used in this study

No.	Mab name (1)	Mab name (2)	Isotype	Reactivity to DENV serotype	Type of Epitope
**1**	D1NS1-84	84B	IgG1	1	conformation
**2**	D2NS1-3D1	3D1	IgG1	1, 2	conformation
**3**	D2NS1-1A4	1A4	IgG2a	2	conformation
**4**	D2NS1-1B10	1B10	IgG1	2	linear
**5**	D2NS1-2C5	2C5	IgM	2	conformation
**6**	D2NS1-4B4	4B4	IgM	2	conformation
**7**	D2NS1-1B2	1B2	IgG1	1-4	linear
**8**	D2NS1-2G6	2G6	IgM	1, 2	conformation
**9**	D2NS1-3A2	3A2	IgG1	2	conformation
**10**	D2NS1-4D2	4D2	IgG1	1-4	linear
**11**	D3NS1-7	7A	IgG2b	3	conformation
**12**	D3NS1-46	46A	IgG2b	3	conformation
**13**	D3NS1-5F3	5F3	IgG1	1, 3	linear
**14**	D4NS1-4	4A	IgG2b	4	conformation
**15**	D4NS1-24	24A	IgG1	4	conformation
**16**	D4NS1-181	181	IgG1	4	conformation
**17**	D4NS1-9	S9.6	IgG1	1, 4	linear
**18**	NS1-1F (2E11)	2E11	IgM	1-4 and flaviviruses	linear
**19**	NS1-3F (1F11)	1F11	IgG2a	1-4	linear
**20**	NS1-4F (2E3)	2E3	IgG1	1-4	linear

(1) Mabs named according to previous publications; No.1-17 (Puttikhunt et al, 2011), No 18-20 (Puttikhunt et al, 2003)

(2) Mabs named as abbreviation in this study.
