# Supplementary material for: High performance dengue virus antigen-based serotyping-NS1-ELISA (plus): A simple alternative approach to identify dengue virus serotypes in acute dengue specimens
Source: PLoS Negl Trop Dis. 2021 Feb 26;15(2):e0009065. doi: 10.1371/journal.pntd.0009065 (PMC7946175; doi:10.1371/journal.pntd.0009065)
Supplement: S2 Table — (PDF) [file pntd.0009065.s005.pdf]

S2 Table. Summary of immune status, severity and dengue serotypes of acute-phase patients' plasma used in this study.

Acute-phase plasma	No. of samples	Serotyping by RT-PCR			
		**DENV1**	**DENV2**	**DENV3**	**DENV4**
**DENV cases (PCR positive)**	**200**	**50**	**55**	**45**	**50**
**Primary infection (DF/DHF)**	4 (2/2)	3 (1/2)	0 (0/0)	1 (1/0)	0 (0/0)
**Secondary infection (DF/DHF)**	196 (76/119)	47 (20/27)	55 (16/38)	44 (19/25)	50 (21/29)
**OFI**	**50**	0	0	0	0
