# Supplementary material for: High performance dengue virus antigen-based serotyping-NS1-ELISA (plus): A simple alternative approach to identify dengue virus serotypes in acute dengue specimens
Source: PLoS Negl Trop Dis. 2021 Feb 26;15(2):e0009065. doi: 10.1371/journal.pntd.0009065 (PMC7946175; doi:10.1371/journal.pntd.0009065)
Supplement: S3 Table — (PDF) [file pntd.0009065.s006.pdf]

S3 Table. Binding kinetics of anti-NS1 Mabs to dengue NS1 antigen by Surface Plasmon Resonance (SPR)

Antibody name	Specificity	rD1NS1			rD2NS1			rD3NS1		
		K_{on} ($10^4 M^{-1}s^{-1}$)	K_{off} ($10^{-4}s^{-1}$)	K_D (nM)	K_{on} ($10^4 M^{-1}s^{-1}$)	K_{off} ($10^{-4}s^{-1}$)	K_D (nM)	K_{on} ($10^4 M^{-1}s^{-1}$)	K_{off} ($10^{-4}s^{-1}$)	K_D (nM)
**1B10**	**D2**				26.10±6.43	8.14±1.01	3.18±0.37			
**3D1**	**D2**				61.70±55.52	11.0±6.22	2.02±0.43			
**1A4**	**D2**				35.00±26.16	16.80±1.97	6.25±4.07			
**4B4**	**D2**				**ND**	**ND**	**ND**			
**5F3**	**D1, D3**	5.53±0.74	2.58±0.48	4.72±1.00				8.37±2.92	1.93±0.46	2.44±0.59
**84B**	**D1**	2.49±1.24	12.3±7	54.8±24.5						
**46A**	**D3**							21.30±16.26	20.50±10.24	12.30±7.00

ND = Unable to determine due to its over ranged values.
